# Decoration of Ultramicrotome-Cut Polymers with Silver Nanoparticles: Effect of Post-Deposition Laser Treatment

**DOI:** 10.3390/ma15248950

**Published:** 2022-12-14

**Authors:** Markéta Kaimlová, Jana Pryjmaková, Miroslav Šlouf, Oleksiy Lyutakov, Giovanni Ceccio, Jiří Vacík, Jakub Siegel

**Affiliations:** 1 Department of Solid State Engineering, University of Chemistry and Technology Prague, 166 28 Prague, Czech Republic; 2 Institute of Macromolecular Chemistry, Academy of Sciences of the Czech Republic, Heyrovského nám. 2, 162 06 Prague, Czech Republic; 3 Department of Neutron Physics, Nuclear Physics Institute (NPI) of the Czech Academy of Sciences (CAS), 250 68 Husinec-Rez, Czech Republic

**Keywords:** optomechanical manipulation, plasmon resonance, silver nanoparticles, polyethylene terephthalate, polyether etherketone, surface morphology

## Abstract

Today, ultramicrotome cutting is a practical tool, which is frequently applied in the preparation of thin polymeric films. One of the advantages of such a technique is the decrease in surface roughness, which enables an effective recording of further morphological changes of polymeric surfaces during their processing. In view of this, we report on ultramicrotome-cut polymers (PET, PEEK) modified by a KrF excimer laser with simultaneous decoration by AgNPs. The samples were immersed into AgNP colloid, in which they were exposed to polarized laser light. As a result, both polymers changed their surface morphology while simultaneously being decorated with AgNPs. KrF laser irradiation of the samples resulted in the formation of ripple-like structures on the surface of PET and worm-like ones in the case of PEEK. Both polymers were homogeneously covered by AgNPs. The selected area of the samples was then irradiated by a violet semiconductor laser from the confocal laser scanning microscope with direct control of the irradiated area. Various techniques, such as AFM, FEGSEM, and CLSM were used to visualize the irradiated area. After irradiation, the reverse pyramid was formed for both types of polymers. PET samples exhibited thicker transparent reverse pyramids, whereas PEEK samples showed thinner brownish ones. We believe that his technique can be effectively used for direct polymer writing or the preparation of stimuli-responsive nanoporous membranes.

## 1. Introduction

Polymeric materials are materials that are currently widely used in general in all industrial areas, primarily due to their excellent workability and long-term stability [1]; however, unmodified polymers often exhibit inappropriate material characteristics (e.g., surface roughness [2] and hydrophobicity/hydrophilicity [3]) for their intended use. Therefore, the aim is to improve their properties for their effective use.

Commercially available thin polymeric foils tend to have inappropriate surface roughness and irregularities are unevenly distributed on the surface, depending on the method of production [2]. In addition, they are often scratched during packaging and storage [4]. To overcome this problem in further processing, we cut the polymeric surface using an ultramicrotome. The microtome is an original mechanical instrument that serves to prepare very thin cuts of biological samples for microscopic examination [5]. However, it has been used effectively to cut, for example, botanical specimens [6] and newly developed polymers [7]. Microtome cuts in the preparation of botanical specimens refer to various parts of botanical tissues, very small sections of wood [5] and plants [6], which serve for histological and pathological studies. Brandão et al. [6] used a microtome to cut the aerial parts of the *Portulaca pilosa* L. plant used in Amazon medicine for the treatment of bacteria-related diseases. Such histological sections were suitable for wound care. The study of polymer–metal interfaces was performed by microtome cutting in the work of Baden et al. [7]. In this case, the thin microtome cut of the sample increased the spatial resolution of the atomic force microscopy-based infrared spectroscopy technique.

Another popular way to improve the properties of polymers is their enrichment with noble metal nanoparticles (NPs). Polymeric materials enriched with noble metal NPs then combine the unique properties of both, which contribute to their broad applications in electronics [8], optics [9], catalysis [10], and medicine [11].

One of the most popular noble metal nanoparticles is silver. Today, they have been effectively anchored into several polymeric materials via (i) covalent chemical bonds [12], (ii) physisorption [13], (iii) particle dispersion in a polymer volume [14], (iv) implantation [15], or optomechanical manipulation [16]. The covalent bond between AgNP and the alginate gelatin hydrogel for wound healing was prepared by Diniz et al. [12]. Covalently boned AgNPs prevented cytotoxicity of the material together with strong antibacterial efficacy, which is important for an effective healing process. Iida et al. [13] modified grapheene nanoplates through plasma in liquid to prepare AgNPs-physisorbed graphene nanoplates. They found that the amount of AgNPs increased with the plasma treatment time. Furthermore, they suggested that the amount of AgNPs might be controlled in a single step. The dispersion of AgNP was the aim of the study published by Hupfeld et al. [14]. They provided highly dispersed plasmonic AgNPs into thermoplastic polyurethane polymers, which was the new method to enable incorporation of color into printed objects by 3D printing. Fahmy et al. [15] created thin films of plasma-polymerized acrylic acid/carbon dioxide copolymers with implanted AgNPs using photoreduction by indirect sunlight and studied their dielectric properties. They found that the presence of Ag^+^ ions or Ag^0^ increased the conductivity of the samples.

A new generation of soft and gentle methods for anchoring AgNPs into polymeric carriers is their optomechanical manipulation. It is one of the soft and gentle methods that do not require the use of a chemical interlayer between the NPs and the polymer carrier [16]. Active NPs are strongly bonded, which minimizes their release into surrounding environments [17]. Therefore, this approach can be considered environmentally friendly [18]. The manipulation with NPs is enabled by the use of polarized laser light, the wavelength of which is fairly close to the local maxima of the localized surface plasmon (LSP) excited in NPs [19]. When such optomechanical manipulation is combined with subsequent irradiation of selected areas of the surface of a thin polymeric microtome cut by different types of laser, new possibilities open up in the field of direct polymer writing or the preparation of stimuli-responsive nanoporous membranes.

Unlike the conventional approach for polymer writing, lithography, where a required pattern must be pre-made in the form of a mask and then transferred onto the polymer carrier, optomechanical manipulation with NPs can create a wide range of patterns in a single step [16]. Furthermore, lithographic methods, such as microcontact printing [20], nanoimprint lithography [21], and self-assembled templates [22], are characterized by the high cost associated with template preparation and, moreover, can only duplicate the pattern.

Current polymeric nanoporous membranes, used in molecular sensing [23] and separation [24], or osmotic energy conversion [25], have limited performance due to the trade-off between pore permeability, scalability, and selectivity. The use of stimuli-responsive ligands that respond to external factors and allow the pores to change shape may overcome this problem, because they are able to regulate the geometrical parameters of the membrane and control the transport processes involved. Ligandized AgNPs can effectively act as stimuli-responsive agents. During the ligandization of NPs, certain coordination interactions (metal–thiolate, metal–carbon, metal–oxygen, and metal–nitrogen) are involved. Due to these interactions, charge transfer occurs at the MNPs–ligand interfaces that can affect the optical, electrical, and magnetic properties of functionalized assemblies [26]. Together with the stimuli-responsive ability of the ligands, it can be utilized for the control and monitoring of transport properties of membranes. For functionalization, several strategies have been developed based on chemical procedures [27]. Taking into account the strong bond [19] formed between nanoparticles and material, optomechanical manipulation is one of the promising ways to plainly anchor such ligandized NPs on the walls of pores.

In this work, we prepared ultramicrotome cuts of polyethylene terephthalate (PET) and polyether etherketone (PEEK). At the same time, we prepared AgNPs in the electrochemical way. Silver NPs were then optomechanically processed, so they formed a homogeneous film over the whole surface of ultramicrotome-treated polymers. After the optomechanical manipulation, the selected part of the polymeric surface was irradiated by the semiconductor laser of the confocal microscope, whereas the laser processing was directly controlled by the setting of the confocal microscope under simultaneous observation in non-confocal TV mode. This method seems to be suitable for direct polymer writing or the preparation of nanoporous membranes with stimuli-responsive agents attached to incorporated nanoparticles. For the preparation of such membranes, the combination of wet chemical treatment of optomechanically immobilized AgNPs may be suggested.

## 2. Materials and Methods

### 2.1. Materials, Apparatus and Procedures

The preparation of silver nanoparticles was performed electrochemically using two Ag electrodes in the form of silver bars (99.99% purity, dimensions 40 × 10 mm, Safina a.s., Czech Republic). The electrodes were immersed in sodium citrate electrolyte (1 mM, 100 mL volume, supplied by Sigma-Aldrich Co., St. Louis, MO, USA) and powered by a DC power supply (voltage 15 V, current 150 mA). The DC voltage was applied to the electrodes for 0.5 h under vigorous magnetic stirring of the electrolyte at room temperature. Subsequently, the silver electrodes were carefully removed and the beaker with the resulting solution was stored for 24 h in darkness to complete the AgNPs formation process. The AgNPs solution was then decanted and filtered for macroscopic impurities removal. For more details on the synthesis procedure, see Ref. [28]. Finally, the concentration of as-synthesized AgNPs colloid was measured by atomic absorption spectrometry (AAS), and the size and shape of AgNPs were determined by transmission electron microscopy (TEM).

**Figure 1:**
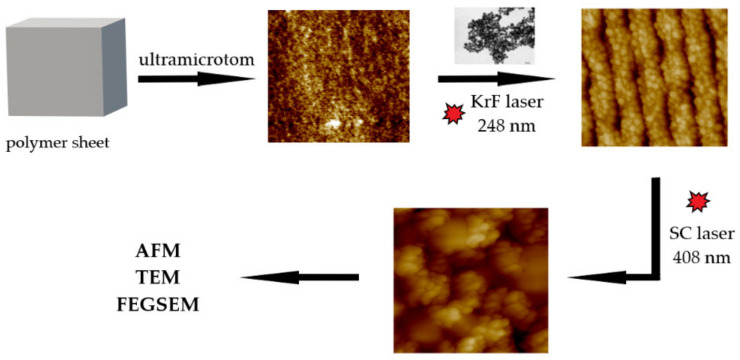
Schematic view of the consecutive steps during sample preparation.

For the incorporation of AgNPs, two polymer sheets, PET and PEEK (thickness of 2 mm, supplied by Goodfellow Ltd., Cambridge, UK), were chosen. Both polymers are capable of changing the surface morphology when irradiated with laser light [19]. Before optomechanical processing, the surface of the polymers was cut with an ultramicrotome (Ultracut UCT, Leica, Viena, Austria), which ensured a very sharp cut (area dimension 2 × 3 mm^2^) that would be substantial for subsequent microscope analysis. Optomechanical processing was performed using a KrF excimer laser (COMPex Pro 50F, Coherent, Inc., Silicon Valley, CA, USA, wavelength 248 nm, pulse duration 20–40 ns, repetition rate 10 Hz, 6000 pulses). Individual polymers were placed vertically and centered in the vertically placed spectroscopic cuvette (Starna Scientific Ltd., Ilford, UK, type 3/Q/100, light path 10 mm). Subsequently, 3 mL of colloidal AgNPs solution was added using an automatic pipette. Subsequently, the samples were irradiated with 6000 laser pulses using a laser fluence of 20 mJ·cm^−2^. Polarized laser light was provided by a linear polarizer (UV-grade fused silica prism, model PBSO-248-100). Irradiation was performed perpendicularly to the PET surface, using an aperture with an area of 5 × 10 mm^2^. The scheme of consecutive steps during sample preparation and characterization is shown in Figure 1. 

After optomechanical processing by KrF excimer laser, the samples were processed on optical microscope using a CLSM confocal laser scanning microscope (Olympus LEXT OLS 3000, Olympus, Sindzuku, Japan) equipped with a violet semiconductor laser (SC, wavelength 408 nm) in confocal mode. Samples processed with 408 nm laser light were simultaneously observed in TV mode using 5× and 10× objective lenses, which provided a total magnification of captured images of 120× and 240×, respectively.

### 2.2. Analytical Methods

The concentration of Ag in as-synthesized Ag colloid was determined by AAS using a flame atomization technique. Measurements were carried out on a Varian AA880 device (Varian Inc., Palo Alto, CA, USA). The uncertainty of the concentration measurement by this method did not exceed 3%. 

AgNP visualization was performed by TEM using JEOL JEM-1010 (JEOL Ltd., Akishima, Japan) operated at 80 kV. The particle size was determined using AnalysSIS 2.0 software, which calculates with at least 500 particles from the TEM micrographs. Before TEM analysis, samples were centrifuged. The AgNPs were then transferred to distilled water. The colloidal solution was dropped on a copper grid coated with a thin amorphous carbon film on filter paper. Before the measurement, the samples were pre-dried by air and stored under vacuum in a desiccator. 

PET and PEEK samples with optomechanically processed AgNPs were characterized by atomic force microscopy (AFM) on a Dimension ICON device (Bruker Corp., Billerica, MA, USA) in ScanAsyst tapping mode in the air. For measurement, an antimony-doped silicon probe type RTESPA-150 was used. The data were evaluated using NanoScope Analysis software.

Another surface visualization method of optomechanically processed samples was a high-resolution field emission gun scanning electron microscopy (FEGSEM) performed on a MAIA3 device (TESCAN, Brno, Czech Republic) equipped with detectors for secondary and backscattered electrons. Measurements were carried out in high-resolution mode at an accelerating voltage of 3 kV.

## 3. Results and Discussion

**Figure 2:**
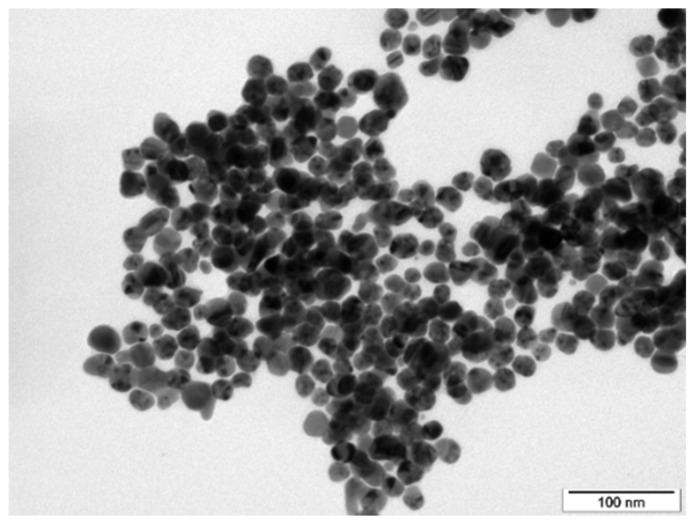
TEM image of electrochemically synthesized AgNPs colloid used for optomechanical processing onto the PEN and PEEK surface.

The shape and morphology of AgNPs in the synthesized colloid solution are obvious from the TEM image (Figure 2). It is evident that the synthesized colloid predominantly contained spherical nanoparticles of narrow size distribution with minimal occurrence of rods. The average size of the NPs was determined at 23.4 ± 4.2 nm, when extreme sizes with a frequency below 1% were eliminated. The concentration of AgNP colloid was determined at 57.4 mg·L^−1^. The colloid of these parameters was used for optomechanical processing onto ultramicrotome-cut PET and PEEK surfaces.

**Figure 3:**
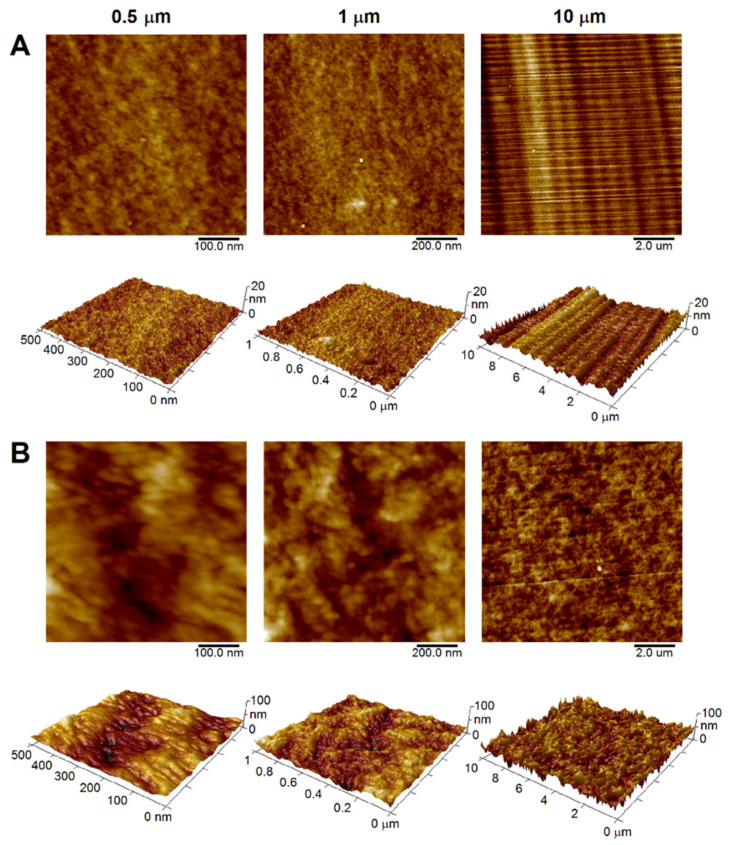
Two-dimensional and three-dimensional AFM scans of ultramicrotome-cut PET (**A**) and PEEK (**B**) surface, scan sizes of 0.5, 1 and 10 µm.

The sharp cut of the polymeric surface (PET, PEEK) by an ultramicrotome was indispensable to ensure a surface as smooth as possible to make subsequent treatment as effective as possible (see below). Table 1 shows that the *R*_a_ values for all three AFM scan sizes achieved considerably low values (around 1 nm for PET and units of nm for PEEK), which is a very promising result compared to commercial polymeric sheets. For example, 50 μm thick PET foil (Goodfellow Ltd., Cambridge, UK) with *R*_a_ values that significantly exceed 1 nm can be mentioned [19]. Compared to commercial foils, one can conclude that the ultramicrotome cut provided regular surface structures (see Figure 3), which lacked irregular unevenness of the surface originating from foil production. This can be confirmed by the low measured SAD values for both polymers examined (Table 1). It is obvious that there are slight differences in the *R*_a_ values between PET and PEEK (slightly higher values for PEEK), which corresponds to the original Goodfellow Ltd. data [29,30], which state that the physical and mechanical properties of these two materials differ slightly (e.g., density 1.30–1.40 vs. 1.26–1.32 g·cm^−3^; tensile modulus 2.00–4.00 vs. 3.70–4.00 GPa for PET and PEEK, respectively). Another parameter that can fundamentally affect the resulting *R*_a_ value is crystallinity, which also differs slightly (max 30% for PET [31], about 35% for PEEK [32]). All these parameters can fundamentally affect the quality of the ultramicrotome cut [33]. The *R*_a_ values then increased with increasing scan size, with the opposite trend of SAD values for both types of polymers, which is well visualized in 3D AFM scans (see Figure 3A,B for PET and PEEK, respectively).

**Figure 4:**
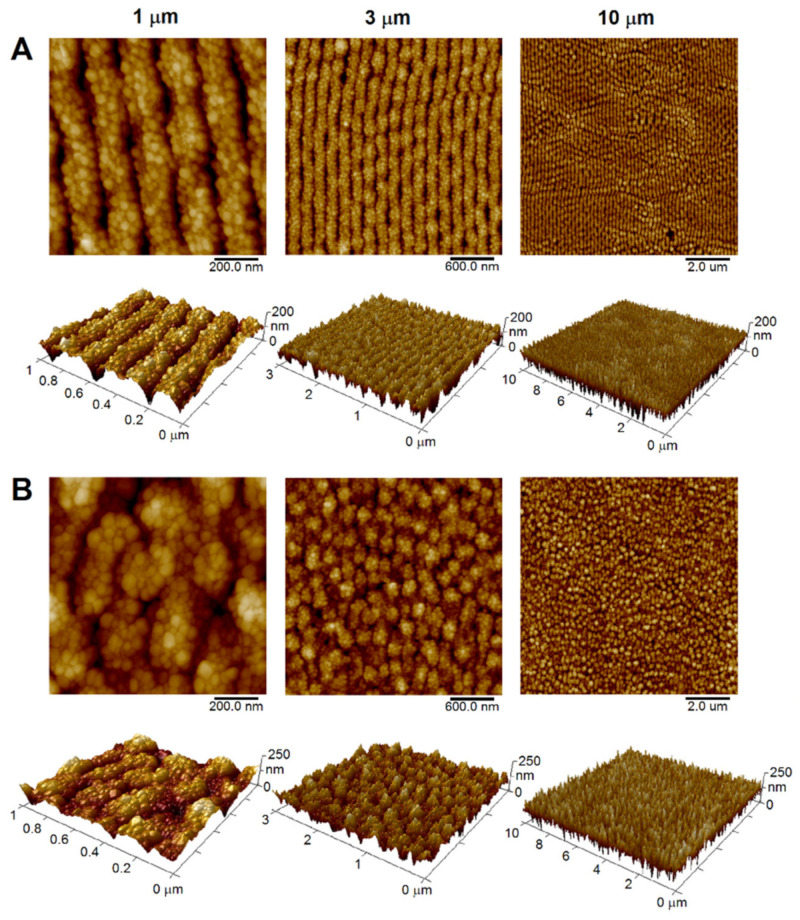
Two-dimensional and three-dimensional AFM images of optomechanically processed AgNPs on PET (**A**) and PEEK (**B**) surface using a KrF laser. Scan sizes of 1, 3 and 10 µm.

Ultramicrotome-cut polymers (PET, PEEK) immersed in the AgNPs colloid were irradiated with a KrF excimer laser, which led to the physical incorporation of AgNPs into their surface (see Figure 4). One can see that exposure of polymers to laser light itself led to the transformation of the surface morphology of the samples (see Figure 3 and Figure 4) into the typical formation of laser-induced periodic surface structures known as LIPSS. Although PET irradiation led to the transformation of the surface structure into ripples (Figure 4A), in case of PEEK the irradiation resulted in worm-like structures (Figure 4B) under the same conditions. AFM images of the highest scan sizes (10 µm) show that LIPSS homogeneously covered all polymer surfaces in the case of PET and PEEK, respectively. These structures are formed as a result of redistribution of polymer matter due to ablation induced by absorbed laser light. Generally, the ability to absorb UV light increases with the presence of conjugated double bonds in its structure [34]. Even though both PET and PEEK contain benzene nuclei in their structure, their chemical structure differs, which may explain the differences between the structures formed by laser light under the same conditions.

Compared to pristine polymers (Table 1), one can see an increase in *R*_a_ values (Table 2 and Table 3 for PET and PEEK, respectively) after KrF laser irradiation. It was predominantly caused by the formation of LIPSS structures with the minor contribution of the presence of AgNPs embedded into the surface. When we compare the values of *R*_a_ for all three scan sizes, one can see that they are practically the same for both polymers, clearly indicating the homogeneous distribution and regularity of LIPSS.

The successful optomechanically driven incorporation of AgNPs into the surface of both types of polymers is clearly apparent in Figure 4 for the lowest scan sizes (1 µm), where one can distinguish individual nanoparticles. From the larger scan sizes (3 and 10 µm), it is evident that AgNPs covered the surface of both polymers regularly. Our approach exploiting polarized laser light results in both surface modification of polymers and embedding of AgNPs into the modified surface in one step. Optomechanical manipulation with AgNPs is enabled due to the presence of localized surface plasmon (LSP). Because of this phenomenon, AgNPs exhibit optical activity, which ensures their thermal excitation by KrF laser light. This type of laser operates at wavelengths close to the LSP resonance maxima of AgNPs [35,36]. Studies [37,38] refer to the fact that spherical AgNPs with a diameter of about 20 nm have LSP resonance maxima around 410 nm. Our study [19] published elsewhere found that during the AgNPs embedding process, the larger particles tend to anchor closer to the polymer surface, while the smaller particles are able to penetrate deeper. The average penetration depth of AgNPs was found to be 70–80 nm.

**Figure 5:**
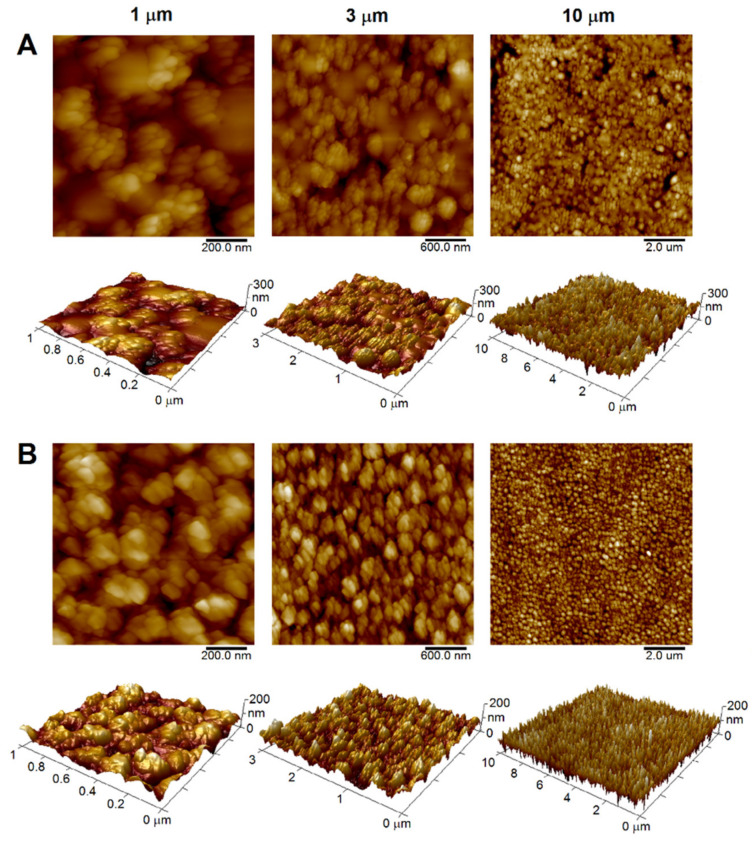
Two-dimensional and three-dimensional AFM scans of PET (**A**) and PEEK (**B**) surfaces with optomechanically processed AgNPs (KrF laser) after their irradiation with SC laser. Images show the center of SC-irradiated area, scan sizes of 1, 3 and 10 µm.

After the immobilization of AgNPs, samples were exposed to the violet semiconductor laser of a confocal microscope in a selected area of their surface. The center part of the Ar-irradiated areas is visualized in Figure 5. Compared to areas of polymer decorated with pure AgNPs (Figure 4), it can be observed that post-deposition 408 nm semiconductor laser irradiation influenced the morphology of LIPSS dramatically. These structures seem to be less regular for both ripples (PET) and worm-like structures (PEEK), respectively. This change was accompanied by a slight decrease in the surface roughness *R*_a_ (see Table 2 and Table 3 for PET and PEEK, respectively). Slight degradation of both types of LIPSS probably occurred. Furthermore, AgNPs that were clearly visible before post-deposition irradiation (Figure 4) were no longer distinguishable on the surfaces of both polymers after irradiation with a 408 nm laser (Figure 5). It is obvious that 408 nm irradiation of polymers led to the additional light-to-heat conversion induced in AgNPs because of the application of the laser light of the wavelength pretty close to LSPR maxima of the incorporated particles. This resulted in local melting and recrystallization of the exposed polymeric area [39], which was manifested by a change in the surface structure of the polymers. These processes probably allowed AgNPs to penetrate deeper into the polymeric surface.

**Figure 6:**
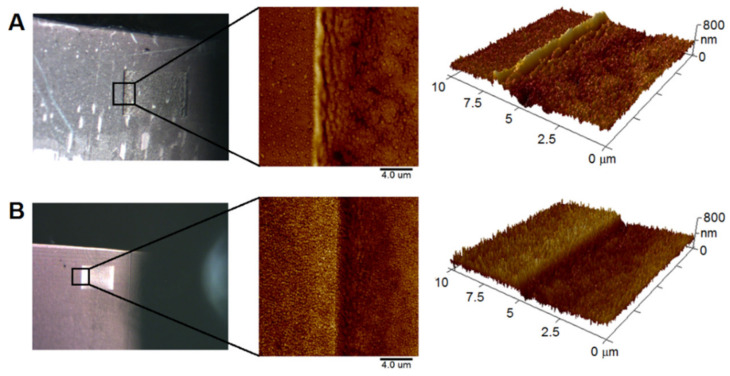
The interface of PET (**A**) and PEEK (**B**) surfaces with optomechanically processed AgNPs after their irradiation with an SC laser. From the left: image from the auxiliary AFM camera; 2D and 3D AFM scans (scan sizes of 10 µm).

Samples processed with a 408 nm laser exhibited a clearly visible interface between unirradiated and irradiated areas, as seen in Figure 6. When observing the samples by AFM with an auxiliary camera (see Figure 6A,B left), one can clearly identify the rectangular area exposed by the 408 nm laser for both polymers (PET and PEEK). Three-dimensional AFM scans of both polymers showed a higher depth of 408 nm irradiated area compared with just AgNPs-decorated polymer surface. This change in surface morphology caused an increase in *R*_a_ values from 20.6 to 51.9 and from 20.0 to 23.2 nm for PET and PEEK, respectively. This change was most noticeable for PET. It was presumably caused by higher thermal conductivity of PET (~0.40 W∙m⁻^1^∙K⁻^1^ [29]) compared to PEEK (~0.25 W∙m⁻^1^∙K⁻^1^ [30]). During the irradiation of the samples by SC laser, the AgNPs were reheated. The reheated NPs then penetrated to the lower depth of the polymers, from which PET was more susceptible to allow the particles to penetrate. The formation of the rectangular area deeper than surroundings after 408 nm laser exposition is also in good accordance with above-mentioned ablation, local melting, and recrystallization of irradiated areas of polymeric surface.

**Figure 7:**
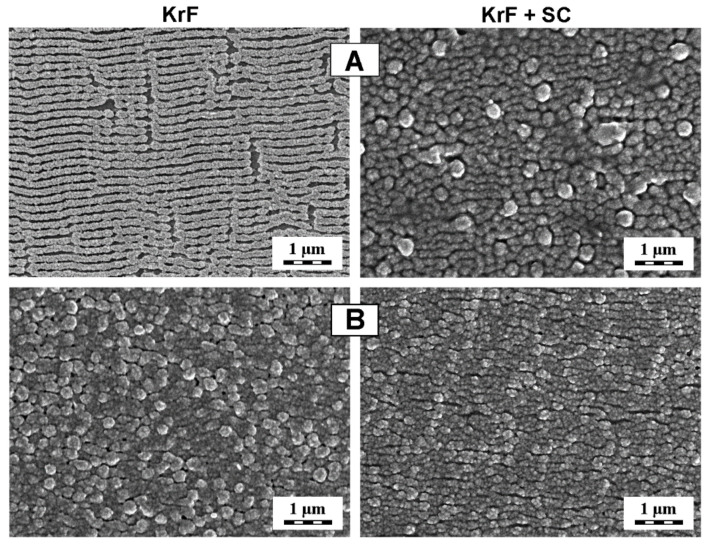
FEGSEM micrographs showing the difference between the surfaces of PET (**A**) and PEEK (**B**) after optomechanical processing of AgNPs (KrF), and subsequent irradiation using SC laser (KrF + SC).

The surface of PET and PEEK samples was then visualized by FEGSEM imaging (see Figure 7). According to the AFM analysis (Figure 4), AgNPs can be observed to be homogeneously distributed on the surface of both types of polymers after their decoration with AgNPs by the KrF laser. After the subsequent irradiation of polymers by a semiconductor 408 nm laser, one can see a noticeable decrease in the amount of detected AgNPs on the surface of both polymers. Most likely, the AgNPs were driven to deeper surface areas of the polymers, and the surfaces reminded many of those of pristine polymers.

**Figure 8:**
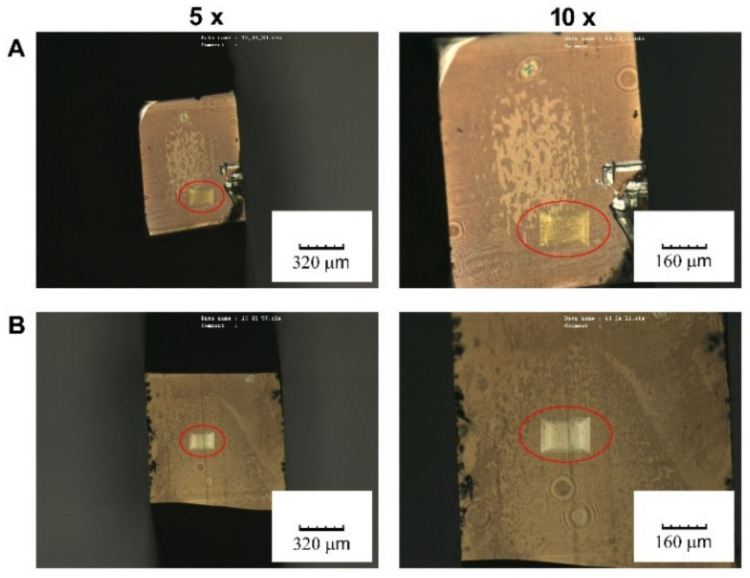
Visualization of the area decorated with AgNPs after its irradiation with SC laser: PET (**A**) and PEEK (**B**). The images were taken by confocal laser scanning microscopy using 5× and 10× objective lenses.

We also examined these 408 nm laser-exposed areas using a confocal microscope in TV mode (see Figure 8). One can observe that the rectangular areas were actually reverse pyramids, the depth of which increased towards the center of SC laser-exposed areas, where the intensity of laser radiation was the highest. On closer examination, one can see that PET samples formed thicker transparent reverse pyramids (Figure 8A), while PEEK samples exhibited thinner brownish pyramids (Figure 8B).

Modification of the PET and PEEK substrates by laser processing of AgNPs colloids changed the properties of the material, which could facilitate applications in various areas of material research. Another benefit introduced by laser processing is the selective change of surface morphology with direct pattern construction [37]. Direct surface patterning may be exploited in direct polymer writing. Regarding the present study, a wide range of patterns can be created in a single step, which is more effective compared to conventional lithography [21], and, moreover, it is cheaper. When optimizing the formation of the reverse pyramid in thin ultramicrotome cut of the polymer, one can effectively prepare nanoporous membranes [27]. Thereafter, stimuli-responsive membranes can be produced by subsequent ligandization of anchored AgNPs.

## 4. Conclusions

We have effectively processed AgNPs of an average size of 23.4 ± 4.2 nm on ultramicrotome-cut PET and PEEK polymeric surfaces by means of optomechanically driven decoration. This procedure caused a simultaneous change in polymeric surface morphology. The surfaces of the polymers differed considerably. While PET exhibited rippled-structures decorated with AgNPs, PEEK revealed worm-like ones. Subsequent post-deposition irradiation of selected polymeric surface area with a semiconductor 408 nm laser of the confocal microscope led to the formation of reverse pyramid-like structures, whereas PET samples formed thicker transparent reverse pyramids, while PEEK exhibited thinner brownish ones. Moreover, the surface structures described above were less regular for both ripples (PET) and wormlike structures (PEEK). In addition, SC laser irradiation caused AgNPs to be driven from the close vicinity of the surface into deeper areas of the polymer as a result of their reheating. The irradiated areas were directly controlled by the TV mode of the confocal microscope used. For this reason, the proposed technology is potentially applicable in direct polymer writing. Future optimization of this process may ensure that reverse pyramid-like structures go through thin ultramicrotome cuts of polymers, which can effectively serve as nanoporous membranes in which functionalized AgNPs could act as stimuli-responsive agents.

## Figures and Tables

**Table 1:** AFM analysis parameters: average surface roughness (*R*_a_) and surface area difference (SAD) for ultramicrotome-cut PET and PEEK samples. The parameters correspond to Figure 2.

Sample	Scan Size (µm)	*R*_a_ (nm)	SAD (%)
	0.5	0.6	4.4
PET (A)	1	0.8	2.1
	10	1.9	0.3
	0.5	4.9	9.2
PEEK (B)	1	4.7	5.3
	10	8.3	2.7

**Table 2:** AFM analysis parameters: average surface roughness (*R*_a_) and surface area difference (SAD) for PET/AgNPs before (PET/AgNPs) and after (PET/AgNPs + SC) 408 nm semiconductor (SC) laser irradiation. The parameters correspond to Figure 4A and Figure 5A.

Sample	Scan Size (µm)	*R*_a_ (nm)	SAD (%)
	1	20.6	89.2
PET/AgNPs	3	21.8	75.2
	10	28.1	49.2
	1	15.1	18.9
PET/AgNPs + SC center	3	20.0	18.0
	10	20.6	15.2

**Table 3:** AFM analysis parameters: average surface roughness (Ra) and surface area difference (SAD) for PEEK/AgNPs before (PEEK/AgNPs) and after (PEEK/AgNPs + SC) 408 nm semiconductor (SC) laser irradiation. Parameters correspond to Figure 3B and Figure 4B.

Sample	Scan Size (µm)	*R*_a_ (nm)	SAD (%)
	1	21.8	67.0
KrF	3	24.0	64.2
	10	26.6	46.9
	1	18.3	42.3
KrF + SC center	3	19.0	25.0
	10	20.0	23.5

## Data Availability

The data presented in this study are available on request from the corresponding author.
